# Socioeconomic inequalities and determinants of maternal health services in Shaanxi Province, Western China

**DOI:** 10.1371/journal.pone.0202129

**Published:** 2018-09-05

**Authors:** Ruo Zhang, Shanshan Li, Chao Li, Doudou Zhao, Leqian Guo, Pengfei Qu, Danmeng Liu, Shaonong Dang, Hong Yan

**Affiliations:** 1 Department of Epidemiology and Biostatistics, School of Public Health, Xi’an Jiaotong University Health Science Center, Xi’an, Shaanxi, People’s Republic of China; 2 Nutrition and Food Safety Engineering Research Center of Shaanxi Province, Xi’an, Shaanxi, People’s Republic of China; La Trobe University, AUSTRALIA

## Abstract

Prenatal health care interventions are effective ways to improve maternal and neonatal health. There have been few large investigations conducted on the inequalities in maternal health services utilization in Shaanxi Province of west China since the health care reform in 2009. This study examined the inequalities and determinants of maternal health services utilization in Shaanxi Province. A household survey was conducted from August to November in 2013. By using a multistage sampling method, local women aged 15–49 who had given birth in the preceding three years were recruited. Information including social-demographic characteristics and maternal health services utilization was collected through a face-to-face interview. A concentration index approach was used to measure inequalities in maternal health services utilization. A logistic regression model was employed to investigate the determinants of maternal health services utilization. There were 8,488 women from urban areas and 18,724 women from rural areas enrolled in this study. The concentration index for all the indicators of maternal health services utilization showed significance in these two areas. In urban areas, the concentration index of having 5 or more prenatal visits, receiving the first prenatal visit within 12 weeks, delivering at secondary- or higher-level health facilities and delivering by C-section were 0.0356, 0.0166, 0.0177 and 0.0591, respectively, while in rural areas, the corresponding figures were 0.0385, 0.0183, 0.0334 and 0.0566, respectively. The determinants related to maternal health services utilization were women’s age at delivery, educational level, employment status, parity, health problems during pregnancy and household income. Inequalities in maternal health services utilization still exist in Shaanxi Province. Providing maternal health services for younger, less educated, unemployed, high parity and poorer women, especially in rural areas, is expected to reduce the inequalities in maternal health services utilization.

## Introduction

Maternal and neonatal health can be improved through the utilization of maternal health services such as prenatal visits [1]. As recommended by the World Health Organization (WHO), all women need access to health services such as prenatal visits, skilled birth attendant and postnatal care visits [2]. Several studies have indicated that skilled health care interventions can reduce the rate of maternal and neonatal mortality by preventing or managing complications during pregnancy and childbirth [3, 4]. However, maternal health services utilization in developing countries, particularly in rural areas, was unsatisfactory [5, 6]. Study in 54 middle-income countries showed that the overall coverage of four or more prenatal visits was only 49.5% [7]. Poverty and lack of health care resources are the main factors that prevent women from receiving adequate health care services during their pregnancy and childbirth [2].

Economic development in China varies significantly across different regions. Compared with the Eastern provinces, economic conditions and health services are relatively underdeveloped in Western provinces [8, 9]. According to the Sixth National Population Census of China in 2010, Shaanxi Province had approximately 37 million people [10]. Previous studies in maternal health services utilization were only conducted in a small number of regions of Shaanxi Province that were of small sample size [8, 11]. The findings from these studies are therefore limited and not able to reveal the overall maternal health services utilization status in Shaanxi Province. The Chinese government carried out health care reform in 2009, with a focus on promoting basic public health services, including allowing urban and rural residents to have equal opportunities to gain access to maternal health services [12]. To the best of our knowledge, there are few, if any, large investigations of the inequalities in maternal health services utilization in Shaanxi Province since the health care reform. In this paper, we aimed to explore the status of inequalities and the determinants of maternal health services utilization in Shaanxi Province. This paper will provide useful insights into the development of effective health care intervention programs and policies in Shaanxi Province.

## Methods

### Design and sample

From August to November of 2013, a cross-sectional study was carried out in Shaanxi Province, Western China. According to the proportion of urban to rural residents in Shaanxi Province, women who had given birth in the preceding three years were enrolled in this study by using a stratified, multistage and random sampling method. The specific details of the sampling method are described elsewhere [13]. Trained skilled investigators (Ph.D. or medical students) and teachers as well as the local health staff in charge of maternal and child health information collection formed the interview teams. By using a structured questionnaire designed by the Xi’an Jiaotong University Health Science Center, information regarding the social-demographic background and status of maternal health services utilization was collected through a face-to-face interview. Each interview lasted for an average of approximately 30 minutes.

### Statistical analysis

Data were double-entered using Epidata version 3.1 (the Epidata association, Odense, Denmark).

For continuous variables, the descriptive data on characteristics of the participants were summarized using mean±SD and compared by a Student *t* test for two groups; for categorical variables, the descriptive data were summarized using counts and proportions and compared by a χ^2^ statistical test.

#### Indicators of maternal health services utilization

According to the recommendation of Chinese Ministry of Health, women should have at least five prenatal visits during pregnancy. In our study, maternal health services utilization was measured in four perspectives: the number of prenatal visits (1: ≥5 prenatal visits; 0: <5 prenatal visits), initiation of the first prenatal visit (1: ≤12 weeks; 0: >12 weeks), place of delivery (1: delivery at secondary- or higher-level health facilities, 0: delivery at health facilities lower than secondary-level), and mode of delivery (1: C-section; 0: vaginal delivery).

#### Measurement of inequalities in the utilization of maternal health services

We used the concentration index (CI) to measure the inequalities in maternal health services utilization. The CI is a measure that quantifies the degree of economic-related inequality in health variable. The CI was proposed by Wagstaff A et al. and was calculated as twice the area between the diagonal and the concentration curve [14]. It can be obtained using the following formula:

$$C=\frac{2}{\mu }cov(h_{i},r_{i})$$

where *C* stands for the concentration index, *h*_*i*_ is the maternal health services utilization index, *μ* is the mean of the maternal health services utilization index, and *r*_*i*_ is the fractional rank of individual annual household income distribution.

The greater the absolute value of the CI, the more the inequality. A value of CI of 0 indicates no inequality in maternal health services utilization. If the CI>0, it means the maternal health services utilization is pro-rich. If the CI<0, it suggests it is pro-poor.

#### Multivariate regression analysis of the determinants in the utilization of maternal health services

The urban and rural classification in this study was defined by the geographical location. We used logistic regression analysis to explore the determinants of maternal health services utilization in urban and rural areas separately. In the data analysis, the following covariates in quantifying the inequalities of maternal health services utilization were considered: maternal age at delivery (<25, 25–29 and ≥30 years old), maternal education (senior high school or higher, junior high school, primary school or lower), maternal employment status (employed or unemployed), parity (1, ≥2), health problems during pregnancy (yes or no) and annual household income (poorest, second poorest, middle, second richest and richest). We report the odds ratio (OR) along with its 95% confidence interval for each of the factors. All data analyses were performed using Stata 12.0 (Stata Corp., Collage Station, TX, USA).

### Ethics statement

This study complied with the Declaration of Helsinki and was approved by the Ethics Committee of Xi’an Jiaotong University Health Science Center (NO. 20120008). Prior to each interview, the consent form was read and explained to each of the participants by the investigators. The interviews were conducted after obtaining the consent form with the participant’s signature or fingerprint (only for participants with apparent low literacy).

## Results

### Baseline characteristics of pregnant women in Shaanxi Province

Table 1 shows the baseline characteristics of the participants classified by area. In total, 8,488 women in urban areas and 18,724 women in rural areas were enrolled in this study, after excluding those who had multiple births (n = 349) and those with missing annual household income (n = 2378). The mean age of the 27,212 women interviewed was 27.0 (SD 4.7) years. Compared with women in urban areas, women in rural areas had a lower proportion of childbirth between 25 to 29 years old (*P*<0.001). These women were less educated and less likely to be employed, and they were also poorer (all *P*<0.001). For the parity, women in rural areas were more likely to have two or more parities compared to their urban counterparts (*P*<0.001). There was no significant difference in the distribution of health problems during pregnancy between the two areas (*P* = 0.064).

**Table 1:** Baseline characteristics of pregnant women by urban and rural areas.

	n	Urban	Rural	*P* value
Number of pregnant women	27212	8488	18724	
Age at delivery, years	27.03±4.76	27.26±4.37	26.93±4.92	<0.001
Age at delivery, years				<0.001
<25	10358	2729 (32.9)	7629 (41.3)	
25–29	10176	3694 (44.5)	6482 (35.1)	
≥30	6260	1878 (22.6)	4382 (23.7)	
Education				<0.001
Senior high school or above	10077	5059 (59.7)	5018 (26.9)	
Junior high school	13714	2967 (35.0)	10747 (57.5)	
Primary school or less	3372	448 (5.3)	2924 (15.6)	
Employment status				<0.001
Employed	8859	4349 (51.6)	4510 (24.2)	
Unemployed	18188	4073 (48.4)	14115 (75.8)	
Parity				<0.001
1	13806	5073 (60.0)	8733 (46.8)	
≥2	13326	3381 (40.0)	9945 (53.2)	
Presence of health problems				0.064
Yes	14027	4307 (51.0)	9720 (52.2)	
No	13020	4134 (49.0)	8886 (47.8)	
Annual household income				<0.001
Poorest	5443	950 (11.2)	4493 (24.0)	
Second poorest	5442	1179 (13.9)	4263 (22.8)	
Middle	5442	1472 (17.3)	3970 (21.2)	
Second richest	5442	2148 (25.3)	3294 (17.6)	
Richest	5443	2739 (32.3)	2704 (14.4)	

### Maternal health care utilization by urban and rural areas

Table 2 describes the differences in the percentage of maternal health services utilization by income groups in urban and rural areas. The proportion of women who had at least 5 prenatal visits in urban areas was significantly higher than that of women from rural areas (81.7% vs. 60.7%, *P*<0.001). Approximately 90% of women in urban areas started their prenatal visits within 12 weeks and delivered at secondary- or higher-level health facilities, while the figures for rural areas were approximately 85% (*P*<0.001, for both). The proportion of women delivered by C-section in rural areas was lower than in urban areas, and the difference was significant (28.7% vs. 44.6%, *P*<0.001).

**Table 2:** Timing and frequency of prenatal visits and delivery care by annual household income in urban and rural areas.

	Annual household income					Total
	Poorest	Second poorest	Middle	Second richest	Richest	
≥5 prenatal visits						
Urban	729 (78.2)	848 (75.8)	1128 (78.9)	1707 (81.6)	2356 (86.9)	6768 (81.7)
Rural	2519 (57.3)	2529 (60.0)	2423 (61.8)	2025 (62.3)	1714 (64.0)	11210 (60.7)
First prenatal visit within 12 weeks						
Urban	797 (86.3)	1005 (87.3)	1296 (89.7)	1928 (91.1)	2474 (91.6)	7500 (89.9)
Rural	3496 (81.4)	3467 (84.0)	3266 (85.0)	2701 (84.6)	2286 (86.4)	15216 (84.1)
Delivery at secondary- or higher-level health facilities						
Urban	831 (88.3)	1062 (90.9)	1364 (93.6)	2019 (94.7)	2602 (96.0)	7878 (93.7)
Rural	3504 (80.1)	3486 (83.7)	3330 (85.5)	2867 (88.9)	2430 (91.1)	15617 (85.2)
C-section						
Urban	407 (43.8)	467 (40.4)	628 (43.5)	920 (43.3)	1302 (48.3)	3724 (44.6)
Rural	1257 (28.3)	1133 (26.8)	1114 (28.3)	992 (30.4)	835 (31.1)	5331 (28.7)

For the distribution of these indicators, an economic gradient was evident in each region. The highest proportion of 5 or more prenatal visits, the first prenatal visit within 12 weeks, the secondary- or higher-level health facilities delivery and the C-section delivery were seen in the richest group in both urban and rural areas.

### Inequalities in maternal health services in urban and rural areas

Table 3 shows the CI with 95% confidence interval for each measurement of the maternal health services utilization in urban and rural areas. Significant inequalities in all the indicators of maternal health services utilization were found in these two areas. The positive value of the CI for these two areas suggests it was pro-rich. The CI of having 5 or more prenatal visits was 0.0356 (95% confidence interval: 0.0299, 0.0416) in urban areas and 0.0385 (95% confidence interval: 0.0320, 0.0454) in rural areas. Similarly, the indicator of receiving the first prenatal visit within 12 weeks shares the same inequality pattern between urban and rural areas. Of these indicators, the CI of delivering at secondary- or higher-level health facilities had the greatest difference between urban and rural areas, and the absolute value of this difference was 0.0157. The strongest economic inequality was seen for C-sections, which was 0.0591 in urban areas and 0.0566 in rural areas.

**Table 3:** The concentration index for maternal health services utilization by urban and rural areas.

	Urban		Rural	
	CI	95% confidence interval	CI	95% confidence interval
≥5 prenatal visits	0.0356*	0.0299, 0.0416	0.0385*	0.0320, 0.0454
First prenatal visit ≤12 weeks	0.0166*	0.0124, 0.0207	0.0183*	0.0147, 0.0220
Delivery at secondary- or higher-level health facilities	0.0177*	0.0145, 0.0209	0.0334*	0.0301, 0.0370
C-section	0.0591*	0.0454, 0.0730	0.0566*	0.0434, 0.0697

* *P*<0.05.

### Multivariate logistic regression of maternal health services utilization in urban areas

Table 4 presents the OR of maternal health services utilization with 95% confidence interval from the logistic regression analysis in urban areas. Compared with women who gave birth before 25 years old, older women were significantly more likely to have 5 or more prenatal visits, a delivery at secondary- or higher-level health facilities and a delivery by C-section. Women with a higher education level were consistently more likely to utilize maternal health services and deliver by C-section than those with primary school or less. For instance, women who completed senior high school or above were 5 times more likely to have 5 or more prenatal visits and 2 times more likely to receive prenatal visits in the first trimester. Our findings specify that employed women were more likely to have adequate prenatal visits (OR = 1.45, 95% confidence interval: 1.27, 1.65) and start their first prenatal visit within 12 weeks (OR = 1.19, 95% confidence interval: 1.01, 1.41). Compared with primipara, women with two or more parities were 30% less likely to have 5 or more prenatal visits, to receive their first prenatal visit within 12 weeks, and to deliver at secondary- or higher-level health facilities as well as were 10% less likely to deliver via C-section. In addition, the presence of health problems during pregnancy had a modest association, with ≥5 prenatal visits (OR = 1.16, 95% confidence interval: 1.03, 1.31). Women from the poorest households were 57% less likely to deliver at secondary- or higher-level health facilities.

**Table 4:** Multivariate logistic regression of maternal health services utilization in urban areas.

	Prenatal Health Care Services		Delivery Outcomes	
Variable	≥5 prenatal visits	First prenatal visit ≤12 weeks	Delivery at secondary- or higher-level health facilities	C-section
	OR (95% confidence interval)	OR (95% confidence interval)	OR (95% confidence interval)	OR (95% confidence interval)
Age at delivery, years				
<25	Ref	Ref	Ref	Ref
25–29	1.31* (1.14, 1.50)	1.15 (0.96, 1.38)	1.32* (1.06, 1.63)	1.17* (1.05, 1.30)
≥30	1.62* (1.36, 1.92)	0.86 (0.70, 1.06)	1.57* (1.21, 2.05)	1.68* (1.48, 1.91)
Education				
Senior high school or above	5.32* (4.25, 6.67)	2.58* (1.97, 3.39)	1.48* (1.01, 2.16)	1.87* (1.50, 2.34)
Junior high school	2.57* (2.07, 3.18)	1.79* (1.38, 2.32)	0.94 (0.65, 1.35)	1.50* (1.21, 1.87)
Primary school or less	Ref	Ref	Ref	Ref
Employment status				
Employed	1.45* (1.27, 1.65)	1.19* (1.01, 1.41)	1.08 (0.88, 1.32)	1.01 (0.91, 1.11)
Unemployed	Ref	Ref	Ref	Ref
Parity				
1	Ref	Ref	Ref	Ref
≥2	0.75* (0.66, 0.86)	0.71* (0.60, 0.84)	0.64* (0.53, 0.79)	0.89* (0.80, 0.99)
Presence of health problems				
Yes	1.16* (1.03, 1.31)	1.14 (0.98, 1.32)	0.91 (0.76, 1.09)	1.09 (1.00, 1.19)
No	Ref	Ref	Ref	Ref
Annual household income				
Poorest	0.96 (0.78, 1.18)	0.81 (0.63, 1.05)	0.43* (0.32, 0.59)	1.03 (0.87, 1.21)
Second poorest	0.82* (0.67, 0.99)	0.83 (0.65, 1.05)	0.57* (0.42, 0.77)	0.86* (0.74, 1.00)
Middle	0.84 (0.70, 1.01)	1.02 (0.81, 1.28)	0.78 (0.58, 1.05)	0.93 (0.81, 1.07)
Second richest	0.82* (0.69, 0.97)	1.07 (0.87, 1.32)	0.83 (0.63, 1.09)	0.87* (0.78, 0.98)
Richest	Ref	Ref	Ref	Ref

* *P*<0.05.

### Multivariate logistic regression of maternal health services utilization in rural areas

Table 5 presents the OR of maternal health services utilization with 95% confidence interval from the logistic regression analysis in rural areas. Older women were more likely to have 5 or more prenatal visits and to deliver by C-section. Women who completed senior high school or above were almost 2 times more likely to have adequate prenatal visits, to receive prenatal visits in the first trimester and to deliver at secondary- or higher-level health facilities than women who had attended primary school or had not attended school. We observed that women’s employment status was only related to mode of delivery, and employed women were 23% more likely to deliver via C-section compared to unemployed women (OR = 1.23, 95% confidence interval: 1.13, 1.33). Women with two or more parities were approximately 30% less likely to have ≥5 prenatal visits, to start their first prenatal visit within 12 weeks, to deliver at secondary- or higher-level health facilities or to deliver by C-section than women with only one parity. These results also indicate that women with health problems during pregnancy were more likely to have 5 or more prenatal visits (OR = 1.23, 95% confidence interval: 1.16, 1.31), to receive their prenatal visits in the first trimester (OR = 1.21, 95% confidence interval: 1.12, 1.32), and to deliver by C-section (OR = 1.24, 95% confidence interval: 1.16, 1.33). Similar to urban areas, rural women from poorer households were consistently less likely to deliver at secondary- or higher-level health facilities. For instance, women from the poorest households were approximately 50% less likely to deliver at secondary- or higher-level health facilities than those from the richest households (OR = 0.51, 95% confidence interval: 0.44, 0.60).

**Table 5:** Multivariate logistic regression of maternal health services utilization in rural areas.

	Prenatal Health Care Services		Delivery Outcomes	
Variable	≥5 prenatal visits	First prenatal visit ≤12 weeks	Delivery at secondary- or higher-level health facilities	C-section
	OR (95% confidence interval)	OR (95% confidence interval)	OR (95% confidence interval)	OR (95% confidence interval)
Age at delivery, years				
<25	Ref	Ref	Ref	Ref
25–29	1.22* (1.13, 1.31)	1.00 (0.90, 1.11)	1.02 (0.92, 1.14)	1.29* (1.19, 1.40)
≥30	1.21* (1.11, 1.33)	0.81* (0.72, 0.91)	0.82* (0.73, 0.93)	1.69* (1.53, 1.87)
Education				
Senior high school or above	2.43* (2.19, 2.69)	1.81* (1.58, 2.07)	1.76* (1.52, 2.04)	1.39* (1.24, 1.56)
Junior high school	1.77* (1.62, 1.93)	1.68* (1.51, 1.86)	1.15* (1.03, 1.28)	1.21* (1.10, 1.34)
Primary school or less	Ref	Ref	Ref	Ref
Employment status				
Employed	1.05 (0.97, 1.14)	1.09 (0.98, 1.22)	1.11 (0.99, 1.25)	1.23* (1.13, 1.33)
Unemployed	Ref	Ref	Ref	Ref
Parity				
1	Ref	Ref	Ref	Ref
≥2	0.69* (0.64, 0.75)	0.64* (0.58, 0.71)	0.74* (0.66, 0.82)	0.73* (0.67, 0.79)
Presence of health problems				
Yes	1.23* (1.16, 1.31)	1.21* (1.12, 1.32)	0.84* (0.77, 0.91)	1.24* (1.16, 1.33)
No	Ref	Ref	Ref	Ref
Annual household income				
Poorest	0.97 (0.87, 1.07)	0.89 (0.77, 1.02)	0.51* (0.44, 0.60)	1.00 (0.90, 1.12)
Second poorest	1.03 (0.93, 1.15)	0.99 (0.86, 1.14)	0.62* (0.53, 0.73)	0.92 (0.82, 1.03)
Middle	1.06 (0.95, 1.18)	1.03 (0.89, 1.19)	0.68* (0.57, 0.80)	0.98 (0.88, 1.10)
Second richest	1.05 (0.94, 1.17)	0.94 (0.81, 1.10)	0.87 (0.73, 1.04)	1.03 (0.92, 1.16)
Richest	Ref	Ref	Ref	Ref

* *P*<0.05.

## Discussion

### The coverage of maternal health services utilization in Shaanxi Province

In this large population-based study, we examined the coverage, disparities, as well as the determinants of maternal health services utilization in urban and rural areas of Shaanxi Province. Our findings showed that the coverages of having adequate prenatal visits, receiving the first prenatal visit within 12 weeks, delivering at secondary- or higher-level institution and delivering by C-section in urban areas were higher than those in rural areas. A study conducted in Southern and Northern areas of China suggested the same finding with our study [15]. The proportions of having adequate prenatal visits, receiving the first prenatal visit within 12 weeks and delivering at secondary- or higher-level health facilities in our study were higher than those in other developing countries such as Ethiopia and Africa, and were also higher than those of previous studies reported in Shaanxi Province and Western China [8, 16–21]. This suggested that the coverage of maternal health services utilization has been improved after years of development, which is consistent with the result of a study comparing the inequality status of maternal health services utilization before and after health care reform in Shaanxi Province[11]. The rates of C-section in our study exceeded the WHO recommended level and were also higher than those from other studies in Bangladesh and African sites [22, 23]. The high prevalence of C-section showed that some surgeries were likely to be clinically unnecessary.

### The inequalities of maternal health services utilization in Shaanxi Province

The main purpose of health care reform is to promote equal basic public health services and to reduce the gap in access to health services in rural and urban areas. The Chinese government has adopted a series of measures, such as implementing subsidies for institutional deliveries in rural areas, to improve maternal and child health care [24]. However, our finding indicated that the inequalities in maternal health services utilization existed in current system and maternal health services utilization was pro-rich both in urban and rural areas, which is consistent with the results reported in studies conducted on a rural area in Western China and two low social economic counties in Shaanxi Province [18, 19]. The CI of having 5 or more prenatal visits and receiving the first prenatal visit within 12 weeks in rural areas were higher than those in urban areas, which indicates the greater inequalities in pre-delivery services utilization in rural areas. This result is also in line with the finding of a study conducted in urban and rural areas of China [25]. We also found that the CI of delivering at secondary- or higher-level health facilities had the greatest difference between urban and rural areas. This might be caused by the unequal allocation of healthcare resources in urban and rural areas. The number of hospital beds per 1,000 people and the number of doctors and nurses per 1,000 people were much higher in urban areas than those in rural areas [26]. The difference could also be explained by the difference between the health care systems among urban and rural areas in China. In rural areas, China implemented the New Rural Cooperative Medical System (NCMS) in 2003. However, the effect of the NCMS on improving poor people's access to formal care was limited [27].

### The determinants of maternal health services utilization in Shaanxi Province

Epidemiological evidence showed that social-demographic characteristics and pregnancy health conditions were factors contributing to maternal health services utilization. We found that, as women had greater age at delivery, they were more likely to have adequate prenatal visits, to deliver at secondary or higher-level health facilities and to deliver by C-section. Previous studies in Central and Eastern China as well as Cambodia also indicated this association [28–30]. Both in urban and rural areas, women with a higher education level were more likely to utilize maternal health services and to deliver by C-section. Similar results were found in the developing countries of Bangladesh, Uganda and Nepal [5, 31, 32]. This outcome may be because highly educated mothers had more knowledge of prenatal visits and tended to realize the benefits of adequate prenatal visits and attendance in the first trimester [33]. On the other hand, some scholars believed that in developing countries, C-section was considered more luxurious than vaginal delivery at the social level [34]. The present study revealed that employed women were more likely to have adequate prenatal visits, to start their first prenatal visit within 12 weeks and to deliver by C-section. These findings were in line with those from studies in South Africa and China [21, 28]. A possible interpretation was the inability of unemployed women to meet the costs associated with prenatal visits and C-section.

In this study, parity was one of the important predictors in determining maternal health services utilization. We found that women with higher parity were significantly less likely to use maternal health services and to choose C-section, which are consistent with other findings [30, 35–38]. These results might be that women with higher parity were more likely to rely on the previous pregnancy experience and felt there was no need to use maternal health services. On the other hand, multipara women may have been more capable of dealing with the difficulties of labor, therefore, they were less likely to choose C-section compared to primipara [39]. One important finding of our study was that women who had pregnancy-related health problems were more likely to have adequate prenatal visits and to deliver by C-section. The result agreed with the Ethiopian and Chinese authors [28, 40, 41]. This could be explained by the fact that the health care utilization during pregnancy often occurred after developing some health problems [40]. Poor economic status was a determinant of inadequate prenatal visits and reduced likelihood of delivering by C-section [42, 43]. Our study also indicated that, both in urban and rural areas, poorer women were less likely to deliver at secondary- or higher-level health facilities. In addition, in urban areas, we found that women with worse economic status were less likely to have adequate prenatal visits and to deliver by C-section.

### Limitation and strengths

The strength of this study is the large sample size. According to the rates of urban to rural residents, the study was conducted by using a multi-stage stratified random sampling method. The inclusion of 27,212 pregnant women makes this study one of the largest studies on this subject in Shaanxi Province. Thus, the results reflected maternal health services utilization in Shaanxi Province and even can be generalized to the other areas in Western China.

It is important to discuss the limitations of our study. First, the data were self-reported by participants, and this may have caused a certain degree of recall bias, although the study showed that the reporting of the number of prenatal visits by women did not differ significantly from the number shown by the medical records [44]. We made efforts to minimize the recall bias; the survey was tested in a pilot study. According to detailed interviewer guides, all the interviewers from the Xi’an Jiaotong University received uniform training before the formal investigation. In addition, standardized questionnaires and detailed supporting materials such as calendars were used to collect the information for the investigation. Second, there were other sources of income, such as household production, that cannot be reflected by annual household income; therefore, it was not completely accurate to use annual household income to measure living standards. Third, owing to the limitation of the pre-specified questions in this survey, we only adjusted for social-demographic characteristics in the multivariate regression analysis. However, other potential confounders and unobservable factors were always of concern in the present study. Nevertheless, as the largest survey in Shaanxi Province, our results provided the status and the determinants of maternal health services utilization in this geographical region.

## Conclusions

In general, the coverage of maternal health services utilization was higher than other regions of China and other developing countries. However, the inequalities in the utilization of maternal health services still exist in urban and rural areas in Shaanxi Province. Providing maternal health services for younger, less educated, unemployed, high parity and poorer women, especially in rural areas, is expected to reduce the inequalities in maternal health services utilization.

## Supporting information

S1 File Survey questions in Chinese. (DOCX)

S2 File Survey questions in English. (DOCX)

S1 Dataset Maternal health services utilization in Shaanxi Province. (SAV)
