# Supplementary material for: Socioeconomic inequalities and determinants of maternal health services in Shaanxi Province, Western China
Source: PLoS One. 2018 Sep 5;13(9):e0202129. doi: 10.1371/journal.pone.0202129 (PMC6124721; doi:10.1371/journal.pone.0202129)
Supplement: S1 File — (DOCX) [file pone.0202129.s001.docx]

**调查问题**

| **县(区)名(County)** |  | **乡镇/街道办名(Township)** |  | **村/社区名(Village)** |  |
| --- | --- | --- | --- | --- | --- |
| **县编码(CC)** |  | **乡编码 (TC)** |  | **村编码 (VC)** |  |
| **妇女编码(PC)** |  | | **妇女联系方式(Tel)** | |  |
| **确认该妇女是否符合要求:**  1.2010年以来,您是否曾经怀孕? ①是 ②否  2.目前,您是否怀孕? ①是 ②否 ③不详  该妇女是否符合调查对象选择标准? ①是 ②否  **若回答”是”,继续本次调查**  调查员姓名: ____________  调查日期: ______________ | | | | | |

**孩子信息**

| **末次怀孕结局(2010年以来,现孕妇女则问上一次怀孕结局)**  ①活产 ②死胎、死产 ③引产  ④人工流产 ⑤药物流产 ⑥自然流产 ⑦其他______  **若末次怀孕结局为活产，且儿童依然生存，则记录其姓名、性别、生日、年龄（若为双胎,请记录另一个孩子姓名、性别）** | | |
| --- | --- | --- |
| C1 | 孩子姓名：________  若为双胎，另一个孩子姓名：_________ | C1a______  C1b______ |
| C2 | 孩子性别：①男 ②女  若为双胎，另一个孩子性别：①男 ②女 | C2a______  C2b______ |
| C3 | 孩子出生日期：____年___月___日 | C3______ |
| C4 | 孩子出生体重：①_____克 ②不详 | C4a______  C4b______ |
| C5 | 胎龄：①_____周+_____天 ②不详 | C5a______  C5b______  C5c______ |
| C6 | 生产方式：①阴道分娩 ②剖宫产 | C6 |

**家庭基本情况**

（应答者为2010-2013年度曾经怀孕的妇女,包括现孕妇女）

| F1 | 妇女姓名：_________ | F1______ |
| --- | --- | --- |
| F2 | 妇女出生日期：_____年___月___日 | F2______ |
| F3 | 妇女民族：①汉 ②回 ③其他______ | F3a______  F3b______ |
| F4 | 妇女文化程度：①大学或大专 ②高中/中专 ③初中 ④小学 ⑤没上过学 | F4______ |
| F5 | 妇女婚姻状况：①初婚 ②再婚 ③离异 ④丧偶 ⑤其他______ | F5a______  F5b______ |
| F6 | 妇女药敏史：①无 ②有______ ③不详 | F6a______  F6b______ |
| F7 | 妇女职业：①农民(种田，副业)或家务 ②工人（具体工种______）  ③民办教师 ④干部、公务员  ⑤商业和服务业从业人员 ⑥科技人员和其他知识分子  ⑦军人 ⑧其他______ | F7a______  F7b______  F7c______ |
| F8 | 丈夫姓名：_________ | F8______ |
| F9 | 丈夫出生日期：_____年___月___日 | F9______ |
| F10 | 丈夫民族：①汉 ②回 ③其他______ | F10a_____  F10b_____ |
| F11 | 丈夫文化程度：①大学或大专 ②高中/中专 ③初中  ④小学 ⑤没上过学 | F11______ |
| F12 | 丈夫婚姻状况：①初婚 ②再婚 ③离异 ④丧偶 ⑤其他 | F12a_____  F12b_____ |
| F13 | 丈夫职业：①农民(种田，副业)或家务 ②工人（具体工种______）  ③民办教师 ④干部、公务员  ⑤商业和服务业从业人员 ⑥科技人员和其他知识分子  ⑦军人 ⑧其他______ | F13a_____  F13b_____  F13c_____ |
| F14 | 您家里有几口人F14a______  其中，子女数F14b______  男孩数F14c______ | F14a_____  F14b_____  F14c_____ |
| F15 | 居民类型：①城镇居民 ②农村居民 | F15______ |
| F16 | 若为城镇户口，  则家庭月收入为F16a______  家庭月支出为F16b______  住房F16c ①自有住房面积______平米 ②租住房屋  汽车F16d ①有（总价格______万元） ②无 | F16a______  F16b_____  F16c_____  F16d_____ |
| F17 | 若为农村户口，则去年农副产品毛收入F17a______元  打工毛收入F17b______元  其他毛收入F17c______元  全年总支出F17d______元  住房类型F17e：①楼房 ②砖瓦房（砖、石窑洞） ③土坯房（土窑）  家用电器（电视、冰箱、洗衣机、空调、电脑）F17f：  ①有____台 ②无  车（可多选）F16g：  ①家用轿车 ②农用车、小面包 ③摩托车、电动车 ④无 | F17a_____  F17b_____  F17c_____  F17d_____  F17e_____  F17f_____  F17g_____ |

**妇女孕期产前检查情况**

| H1 | 怀最后一个孩子时是否做过产前检查  ①是 次 ②否 | H1a______  H1b______ |
| --- | --- | --- |
| H2 | 怀最后一个孩子时是否进行过优生优育咨询  ①是 次 ②否 | H2a______  H2b______ |
| H3 | 怀最后一个孩子时是否进行过产前诊断（主要指胎儿染色体异常的细胞遗传学产前诊断）  ①______诊断 ②否 | H3a______  H3b______ |
| H4 | 第一次产前检查是在怀孕第 个月 | H4______ |
| H5 | 主要在哪进行产前检查：  ①县（区）或县（区）级以上医院  ②县（区）或县（区）级以上妇幼保健院  ③乡镇卫生院（社区卫生服务中心）  ④村卫生室 ⑤计生站 ⑥私人诊所 ⑦其它______ | H5a______  H5b______ |

**妇女孕前3个月-孕期患病情况（MD）**

|  |  | 患病时间（可多选） |  |
| --- | --- | --- | --- |
| MD1 | 感冒 | 1.孕前1-3月 2.孕1-3月 3.孕4-6月 4.孕7-10月 5.未患过 6.不详 | MD1______ |
| MD2 | 发烧 | 1.孕前1-3月 2.孕1-3月 3.孕4-6月 4.孕7-10月 5.未患过 6.不详 | MD2______ |
| MD3 | 妇科疾病^*^ | 1.孕前1-3月 2.孕1-3月 3.孕4-6月 4.孕7-10月 5.未患过 6.不详 | MD3______ |
| MD4 | 妊高征 | 1.孕前1-3月 2.孕1-3月 3.孕4-6月 4.孕7-10月 5.未患过 6.不详 | MD4______ |
| MD5 | 泌尿系感染 | 1.孕前1-3月 2.孕1-3月 3.孕4-6月 4.孕7-10月 5.未患过 6.不详 | MD5______ |
| MD6 | 贫血 | 1.孕前1-3月 2.孕1-3月 3.孕4-6月 4.孕7-10月 5.未患过 6.不详 | MD6______ |
| MD7 | 病毒性肝炎 | 1.孕前1-3月 2.孕1-3月 3.孕4-6月 4.孕7-10月 5.未患过 6.不详 | MD7______ |
| MD8 | 妊娠急性脂肪肝 | 1.孕前1-3月 2.孕1-3月 3.孕4-6月 4.孕7-10月 5.未患过 6.不详 | MD8______ |
| MD9 | 妊娠期肝内胆汁淤积症 | 1.孕前1-3月 2.孕1-3月 3.孕4-6月 4.孕7-10月 5.未患过 6.不详 | MD9______ |
| MD10 | 甲亢 | 1.孕前1-3月 2.孕1-3月 3.孕4-6月 4.孕7-10月 5.未患过 6.不详 | MD10______ |
| MD11 | 甲低 | 1.孕前1-3月 2.孕1-3月 3.孕4-6月 4.孕7-10月 5.未患过 6.不详 | MD11______ |
| MD12 | 糖尿病 | 1.孕前1-3月 2.孕1-3月 3.孕4-6月 4.孕7-10月 5.未患过 6.不详 | MD12______ |
| MD13 | 合并性传播疾病^*^ | 1.孕前1-3月 2.孕1-3月 3.孕4-6月 4.孕7-10月 5.未患过 6.不详 | MD13______ |
| MD14 | TORCH感染—风疹病毒 | 1.孕前1-3月 2.孕1-3月 3.孕4-6月 4.孕7-10月 5.未患过 6.不详 | MD14______ |
| MD15 | TORCH感染—巨细胞病毒 | 1.孕前1-3月 2.孕1-3月 3.孕4-6月 4.孕7-10月 5.未患过 6.不详 | MD15______ |
| MD16 | TORCH感染—弓形虫 | 1.孕前1-3月 2.孕1-3月 3.孕4-6月 4.孕7-10月 5.未患过 6.不详 | MD16______ |
| MD17 | TORCH感染—单纯疱疹病毒I型 | 1.孕前1-3月 2.孕1-3月 3.孕4-6月 4.孕7-10月 5.未患过 6.不详 | MD17______ |
| MD18 | TORCH感染—单纯疱疹病毒II型 | 1.孕前1-3月 2.孕1-3月 3.孕4-6月 4.孕7-10月 5.未患过 6.不详 | MD18______ |
| MD19 | 其他 | 1.孕前1-3月 2.孕1-3月 3.孕4-6月 4.孕7-10月 5.未患过 6.不详 | MD19______ |
| 备注：*妇科疾病：指生殖系统感染，包括阴道炎、附件炎、宫颈炎、真菌感染、霉菌感染等。*  *合并性传播疾病：包括衣原体、支原体、尖锐湿疣、淋病、梅毒、艾滋病等。*  患病（总结以上表格）： ① 是 ② 否 ③ 不详 | | | |
