# Supplementary material for: Socioeconomic inequalities and determinants of maternal health services in Shaanxi Province, Western China
Source: PLoS One. 2018 Sep 5;13(9):e0202129. doi: 10.1371/journal.pone.0202129 (PMC6124721; doi:10.1371/journal.pone.0202129)
Supplement: S2 File — (DOCX) [file pone.0202129.s002.docx]

**Survey Questions**

| County/District |  | Township/Street |  | Village/Community |  |
| --- | --- | --- | --- | --- | --- |
| County Code (CC) |  | Township Code (TC) |  | Village Code (VC) |  |
| Personal Code (PC) |  | | Women’s Telephone Number | |  |
| Please confirm whether the woman meets the requirements.  1. Have you ever been pregnant since 2010? ①Yes ②No  2. Are you pregnant currently? ①Yes ②No ③Unknown  Does the woman meet the survey selection criteria? ①Yes ②No  If you answer "yes", continue with this survey.  Investigator’s Name: ____________  Survey Date: __________________ | | | | | |

**Child information**

Please write your answer in the space.

| **The most recent pregnancy outcome since 2010**  ①Live birth ②Stillbirth ③ Induction of labor ④ Abortion ⑤ Drug abortion  ⑥Natural abortion ⑦Others ______  If the last pregnancy outcome is a live birth and the child is still alive, record the name, sex, birthday, birth weight, gestational age and delivery method (if twins, record the other child's name, gender) | | |
| --- | --- | --- |
| C1 | Child’s name: _________  If twins, please record the other child’s name: _________ | C1a______  C1b______ |
| C2 | Child’s gender: ①Male ②Female  If twins, please record the other child’s gender: ①Male ②Female | C2a______  C2b______ |
| C3 | Child’s birthday: __________ (MM/DD/YYYY) | C3______ |
| C4 | Child’s birth weight: ①_____ grams ②Unknown | C4a______  C4b______ |
| C5 | Gestational age: ①_____weeks and _____days ②Unknown | C5a______  C5b______  C5c______ |
| C6 | Delivery method: ①Vaginal delivery ②C-section | C6 |

**Family basic information**

| F1 | Women’s name: __________ | F1______ |
| --- | --- | --- |
| F2 | Women’s birthday: __________ (MM/DD/YYYY) | F2______ |
| F3 | Women’s ethnic: ①Han ②Hui ③Others______ | F3a______  F3b______ |
| F4 | Women’s educational Level:  ①College degree or above ②High school ③Middle School  ④Primary School ⑤Never attend school | F4______ |
| F5 | Women’s marital status:  ①First marriage ②Remarriage ③Divorced ④Widowed  ⑤Others______ | F5a______  F5b______ |
| F6 | History of drug allergy: ①No ②Yes______ ③Unclear | F6a______  F6b______ |
| F7 | Women’s occupation:  ①Farmer ②Worker ③Teacher ④Civil servant  ⑤Business and Service Workers ⑥Scientific and technical personnel  ⑦Military man ⑧Others______ | F7a______  F7b______ |
| F8 | Spouse’s name: _________ | F8______ |
| F9 | Spouse’s birthday: __________ (MM/DD/YYYY) | F9______ |
| F10 | Spouse’s ethnic: ①Han ②Hui ③Others______ | F10a______  F10b______ |
| F11 | Spouse’s Educational Level:  ①College degree or above ②High school ③Middle School  ④Primary School ⑤Never attend school | F11______ |
| F12 | Spouse’s marital status:  ①First marriage ②Remarriage ③Divorced ④Widowed  ⑤Others______ | F12a______  F12b______ |
| F13 | Spouse’s occupation:  ①Farmer ②Worker ③Teacher ④Civil servant  ⑤Business and Service Workers ⑥Scientific and technical personnel  ⑦Military man ⑧Others______ | F13a______  F13b______ |
| F14 | How many people are there in your family? ______  The number of children ______  The number of boys ______ | F14a______  F14b______  F14c______ |
| F15 | Type of resident: ①Urban resident ②Rural resident | F15______ |
| F16 | If you are a urban resident，  The monthly household income is ______ RMB;  The monthly household expenditure is ______ RMB;  Housing type: ①Own. The housing area is about___square meters ②Rent  Do you own a car? ①Yes. The value is about ______RMB ②No | F16a______  F16b______  F16c______  F16d______ |
| F17 | If you are a rural resident,  The annual agricultural gross income is ______ RMB;  The annual labor gross income is ______ RMB;  The annual other income is ______ RMB;  The Total annual expenditure is ______ RMB;  Housing type: ①Building ②Single-storey house ③Adobe house  Household appliances (such as TV, fridge, washing machine, air conditioning,  Computer): ①Total amount____ ②None  Vehicle information (Multiple choice questions):  ①Car ② Agricultural vehicles ③Motorcycle or Electric car ④None | F17a______  F17b______  F17c______  F17d______  F17e______  F17f______  F17g_____ |

**Prenatal health care during pregnancy**

| H1 | Did you have prenatal visit during the last pregnancy? If the answer is yes, please answer how many times.  ①Yes______ ②No | H1a______  H1b______ |
| --- | --- | --- |
| H2 | Did you have eugenics counseling during the last pregnancy? If the answer is yes, please answer how many times.  ①Yes______ ②No | H2a______  H2b______ |
| H3 | Did you perform prenatal diagnosis during the last pregnancy? If the answer is Yes, please telling us what the diagnosis is.  ①Yes______ ②No | H3a______  H3b______ |
| H4 | When you was pregnant for ______ months, you had your first prenatal visit. | H4______ |
| H5 | Where do you do the prenatal visits mainly?  ①County (District) - or higher- level health facilities  ②County (District) - or higher- level Maternal and Child health care Institution  ③Township hospital (Community service center)  ④Village clinic ⑤Family planning station  ⑥Private clinic ⑦Others______ | H5a______  H5b______ |

**Maternal disease during pregnancy**

Please write your answer in the space on the right.

|  |  | Disease period (Multiple choices) |  |
| --- | --- | --- | --- |
| MD1 | Cold | ①1-3 months before pregnancy ②1-3 months after pregnancy ③4-6 months after pregnancy  ④1-3 months after pregnancy ⑤Not suffering from disease ⑥Unclear | MD1______ |
| MD2 | Fever | ①1-3 months before pregnancy ②1-3 months after pregnancy ③4-6 months after pregnancy  ④1-3 months after pregnancy ⑤Not suffering from disease ⑥Unclear | MD2______ |
| MD3 | Gynecological diseases^*^ | ①1-3 months before pregnancy ②1-3 months after pregnancy ③4-6 months after pregnancy  ④1-3 months after pregnancy ⑤Not suffering from disease ⑥Unclear | MD3______ |
| MD4 | Pregnancy-induced hypertension | ①1-3 months before pregnancy ②1-3 months after pregnancy ③4-6 months after pregnancy  ④1-3 months after pregnancy ⑤Not suffering from disease ⑥Unclear | MD4______ |
| MD5 | Urinary tract infection | ①1-3 months before pregnancy ②1-3 months after pregnancy ③4-6 months after pregnancy  ④1-3 months after pregnancy ⑤Not suffering from disease ⑥Unclear | MD5______ |
| MD6 | Anemia | ①1-3 months before pregnancy ②1-3 months after pregnancy ③4-6 months after pregnancy  ④1-3 months after pregnancy ⑤Not suffering from disease ⑥Unclear | MD6______ |
| MD7 | Viral hepatitis | ①1-3 months before pregnancy ②1-3 months after pregnancy ③4-6 months after pregnancy  ④1-3 months after pregnancy ⑤Not suffering from disease ⑥Unclear | MD7______ |
| MD8 | Acute fatty liver of pregnancy | ①1-3 months before pregnancy ②1-3 months after pregnancy ③4-6 months after pregnancy  ④1-3 months after pregnancy ⑤Not suffering from disease ⑥Unclear | MD8______ |
| MD9 | Intrahepatic cholestasis during pregnancy | ①1-3 months before pregnancy ②1-3 months after pregnancy ③4-6 months after pregnancy  ④1-3 months after pregnancy ⑤Not suffering from disease ⑥Unclear | MD9______ |
| MD10 | Hyperthyroidism | ①1-3 months before pregnancy ②1-3 months after pregnancy ③4-6 months after pregnancy  ④1-3 months after pregnancy ⑤Not suffering from disease ⑥Unclear | MD10______ |
| MD11 | Hypothyroidism | ①1-3 months before pregnancy ②1-3 months after pregnancy ③4-6 months after pregnancy  ④1-3 months after pregnancy ⑤Not suffering from disease ⑥Unclear | MD11______ |
| MD12 | Diabetes | ①1-3 months before pregnancy ②1-3 months after pregnancy ③4-6 months after pregnancy  ④1-3 months after pregnancy ⑤Not suffering from disease ⑥Unclear | MD12______ |
| MD13 | Combined sexually transmitted diseases^*^ | ①1-3 months before pregnancy ②1-3 months after pregnancy ③4-6 months after pregnancy  ④1-3 months after pregnancy ⑤Not suffering from disease ⑥Unclear | MD13______ |
| MD14 | TORCH infection--Rubella virus | ①1-3 months before pregnancy ②1-3 months after pregnancy ③4-6 months after pregnancy  ④1-3 months after pregnancy ⑤Not suffering from disease ⑥Unclear | MD14______ |
| MD15 | TORCH infection--Cytomegalovirus | ①1-3 months before pregnancy ②1-3 months after pregnancy ③4-6 months after pregnancy  ④1-3 months after pregnancy ⑤Not suffering from disease ⑥Unclear | MD15______ |
| MD16 | TORCH infection--Toxoplasma gondii | ①1-3 months before pregnancy ②1-3 months after pregnancy ③4-6 months after pregnancy  ④1-3 months after pregnancy ⑤Not suffering from disease ⑥Unclear | MD16______ |
| MD17 | TORCH infection--Herpes simplex virus type I | ①1-3 months before pregnancy ②1-3 months after pregnancy ③4-6 months after pregnancy  ④1-3 months after pregnancy ⑤Not suffering from disease ⑥Unclear | MD17______ |
| MD18 | TORCH infection--Herpes simplex virus type II | ①1-3 months before pregnancy ②1-3 months after pregnancy ③4-6 months after pregnancy  ④1-3 months after pregnancy ⑤Not suffering from disease ⑥Unclear | MD18______ |
| MD19 | Others | ①1-3 months before pregnancy ②1-3 months after pregnancy ③4-6 months after pregnancy  ④1-3 months after pregnancy ⑤Not suffering from disease ⑥Unclear | MD19______ |
| Remarks:  ①Gynecological diseases: refers to reproductive system infections, including vaginitis, attachment inflammation, cervicitis, fungal infections, and fungal infections.  ②Sexually transmitted diseases include: chlamydia, mycoplasma, condyloma, gonorrhea, syphilis, and AIDS.  **Summary**  Whether suffering from disease during pregnancy? ①Yes ②No ③Unclear | | | |
